# Single B-Cell-Based Generation of Porcine Anti-CSFV E^rns^ Monoclonal Antibodies and Application in a Blocking ELISA Assay

**DOI:** 10.3390/ijms27114993

**Published:** 2026-05-30

**Authors:** Yufeng Huang, Jiaxin Li, Fangtao Li, Junjie Zhao, Lu Xu, Xingqi Zou, Qi Li, Junfeng Zhu, Yan Li, Yingju Xia, Yebing Liu, Qizu Zhao, Yuanyuan Zhu

**Affiliations:** National/WOAH Reference Laboratory for Classical Swine Fever, China Institute of Veterinary Drug Control, Beijing 100081, China; huangyf0522@163.com (Y.H.); lijiaxinswq@163.com (J.L.); zhaojunjie_ivdc@163.com (J.Z.); xulu777_ivdc@163.com (L.X.); zouxingqi@163.com (X.Z.); 18201364967@163.com (Q.L.); zjf15615953692@outlook.com (J.Z.); liyannongda@foxmail.com (Y.L.); vet_xiayj@163.com (Y.X.); zjsliuyebing@163.com (Y.L.); zhaoqizu@163.com (Q.Z.)

**Keywords:** classical swine fever virus (CSFV), E^rns^, single B cell, monoclonal antibody, blocking ELISA

## Abstract

Classical swine fever (CSF), caused by the classical swine fever virus (CSFV), is an acute, febrile, and highly contagious disease that has led to significant economic losses in the global swine industry. Although the attenuated lapinized CSF vaccine (C-strain) has effectively controlled CSF outbreaks in China since the 1950s, it remains challenging to serologically differentiate infected from vaccinated animals (DIVA). Currently, the application of E2 subunit vaccines allows for DIVA by detecting antibodies against the E^rns^ protein. Therefore, this study aimed to develop a blocking ELISA for CSFV E^rns^ antibody detection using porcine monoclonal antibodies (mAbs) derived from single B cell technology. Peripheral blood mononuclear cells (PBMCs) were isolated from immunized pigs, and single CD21^+^IgM^−^E^rns^-His tag^+^ B cells were sorted via flow cytometry. Using one-step PCR, full-length genes of porcine IgG heavy and light chains were amplified separately, yielding 11 porcine mAbs against the CSFV E^rns^ protein. Among these, three mAbs (E0S3, E0S5, and E0S10) exhibited broad reactivity, while two (E0S1, E0S4) showed no cross-reaction with bovine viral diarrhea virus (BVDV). Using mAb E0S4 as the blocking antibody, a blocking ELISA was established and optimized. The assay demonstrated a detection limit of 1:128, no cross-reactivity with other swine viruses or BVDV, and intra- and inter-assay coefficients of variation below 10%. ROC curve analysis determined an optimal cut-off value of 48.4%, with high sensitivity and specificity. In conclusion, the developed blocking ELISA provides a reliable tool for high-throughput serological surveillance, facilitating the DIVA strategy and contributing to CSF eradication programs.

## 1. Introduction

Classical swine fever (CSF), a highly contagious viral disease of swine, continues to pose significant economic threats to the global pork industry [1]. The causative agent, classical swine fever virus (CSFV), belonging to the genus *Pestivirus* within the family *Flaviviridae*, exhibits antigenic similarities with other members of *pestiviruses* such as bovine viral diarrhea virus (BVDV). This homology often results in serological cross-reactivity, complicating accurate diagnosis and surveillance efforts [2,3,4]. The lapinized C-strain vaccine, developed in the mid-20th century by China, has been pivotal in controlling CSF epidemics in many endemic regions due to its exceptional efficacy and safety profile [5,6]. However, a major limitation of conventional live attenuated vaccines like the C-strain is the inability to serologically differentiate infected from vaccinated animals (DIVA) [7,8], which is a critical requirement for disease eradication campaigns and international trade.

The CSFV genome encodes two major immunogenic structural glycoproteins, E2 and E^rns^ [9,10,11]. While both elicit neutralizing antibodies, E2 is the primary target for most commercially available vaccines and diagnostic assays, due to its high conservation across strains, presence of critical glycosylation sites, and immunodominance in eliciting potent neutralizing antibodies [12,13]. The recent widespread deployment of E2 subunit vaccines has further exacerbated the diagnostic dilemma, as tests based on E2 cannot distinguish between subunit vaccine recipients and animals infected with wild-type virus [14,15]. In contrast, antibodies against E^rns^ are typically produced during natural infection but not by E2 subunit vaccines, making E^rns^ an ideal target for developing DIVA-compliant serological tests [16,17].

Single B-cell antibody technology, an emerging and powerful approach, enables efficient isolation of antigen-specific B cells and amplification of antibody genes, thereby overcoming limitations of traditional methods like hybridoma technology [18,19]. A key advantage of this platform is its broad species adaptability, allowing for the flexible generation of monoclonal antibodies (mAbs) from various immunized host animals, including pigs, rabbits, and mice, as required by the research objectives. Given that pigs are the natural host for CSFV, porcine-derived mAbs are likely to exhibit superior affinity and reflect a more authentic antigenic response profile compared to those from murine systems, potentially enhancing diagnostic performance. Supporting this approach, Wang et al. [20] and Li et al. [21] successfully cloned IgG heavy (H) chains and light (L) chains from porcine B cells using one-step PCR and two-step PCR methods, respectively. Furthermore, by applying a microfluidics-based high-throughput single B cell platform, Liu et al. [22] recently identified and characterized a pool of monoclonal antibodies (mAbs) targeting the B/C domain of the CSFV E2 protein. Their work yielded 82 distinct, naturally paired antibody sequences via next-generation sequencing, revealing mAbs with distinct cross-reactivity profiles and novel pan-pestivirus epitopes. This underscores the power of the technology in porcine diagnostic tools and marker vaccines.

Therefore, this study aimed to leverage single B-cell antibody technology to generate a panel of whole-porcine mAbs against CSFV E^rns^ protein. Subsequently, we sought to develop and validate a blocking ELISA for the specific detection of E^rns^ antibodies, thereby providing a robust and reliable serological tool to support CSF surveillance and advance eradication programs.

## 2. Results

### 2.1. Isolation of Anti-E^rns^-Specific Porcine B Cells

**Figure 1:**
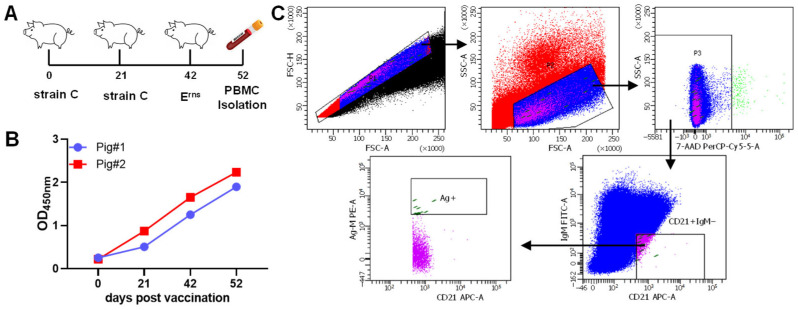
Isolation of Anti-E^rns^-Specific Porcine B Cells. (**A**) Immunization schedule of pigs. For the first and second immunizations, each pig received 10 doses of classical swine fever C-strain vaccine via cervical muscle injection. Forty-two days post vaccination (dpv), a booster immunization with purified CSFV E^rns^ protein was administered at a dose of 100 μg per pig through the same route. PBMC were isolated on 52 dpv. (**B**) Detection of anti-E**^rns^** antibody titers in serum samples collected at 0, 21, 42, and 52 dpv. CSFV E^rns^ positive serum and SPF pig serum were used as positive and negative controls, respectively. (**C**) Distribution and proportion of CD21^+^IgM^−^E^rns^-His tag^+^ porcine B cells in peripheral blood of pig#2 as determined by FACS.

Pigs were immunized with two doses of the C-strain vaccine followed by a booster of purified E^rns^ protein. The anti-E^rns^ antibody response was then assessed by indirect ELISA at 52 days post the initial vaccination. As we expected, both pigs generated a very high level of antibody responses against the E^rns^ protein (Figure 1A,B). PBMCs were isolated from the pig with the higher antibody titer and subjected to fluorescence-activated cell sorting (FACS) to identify anti-E^rns^-specific B cells. The gating strategy is illustrated in Figure 1C. Single cells were first selected, followed by gating on the lymphocyte population and exclusion of dead cells. B lymphocytes were identified as CD21^+^ and IgM^−^. Finally, anti-E^rns^-specific B cells (CD21^+^IgM^−^E^rns^-His tag^+^) were gated based on the His tag. A total of 48 target single B cells were sorted at single-cell density into a 96-well PCR plate for downstream single B cell IgG gene-specific PCR reactions.

### 2.2. Amplification of IgG Heavy and Light Chains from Porcine Single B Cell

**Figure 2:**
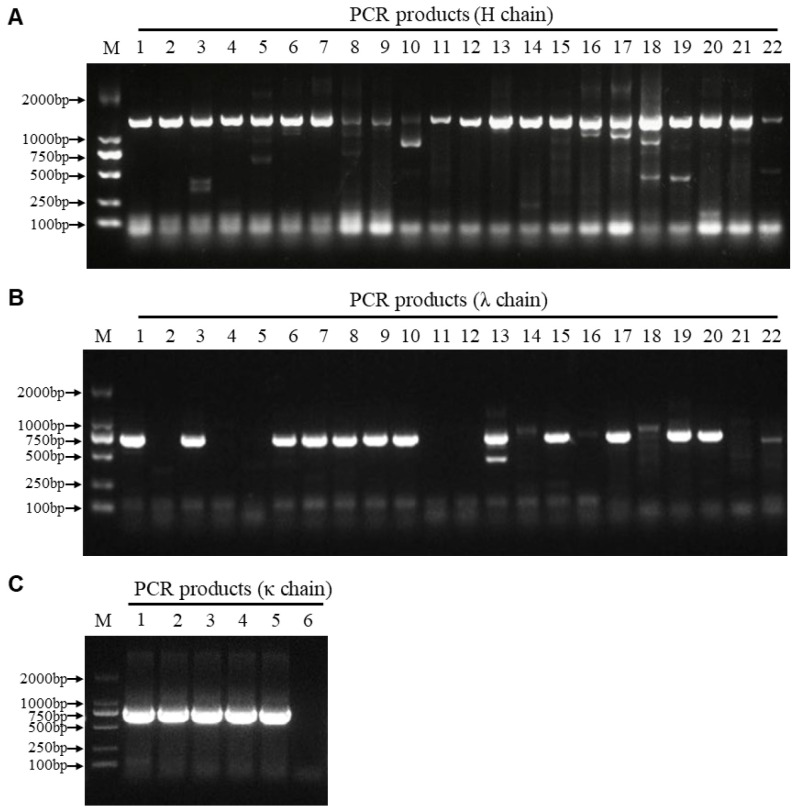
Single B-cell PCR to amplify the whole coding regions of porcine IgG chains. (**A**–**C**) 1% gel electrophoresis patterns of the PCR products for heavy chains (**A**), λ light chains (**B**) and κ light chains (**C**).

To efficiently amplify the whole coding regions of porcine IgG chains, we designed degenerate primers based on the available full-length porcine IgG gene sequences in GenBank (Table 1). The PCR products showed the expected size upon agarose gel electrophoresis analysis. Distinct bands were observed at approximately 1400 bp for heavy chains (Figure 2A) and around 650 bp for light chains (Figure 2B,C). From 48 sorted single B cells, we successfully obtained 20 IgG H chains, 12 lambda (λ) light chains and 5 kappa (κ) light chains. Of these, 11 constituted naturally paired heavy and light chain sets (E0S1 to E0S11), since each set was amplified from a common single-cell well, thereby preserving their native pairing.

Sequence analysis of the variable regions of heavy and light chains using the IMGT^®^ database revealed diverse V(D)J gene usage (Table 2). Among the 11 paired antibody genes, seven consisted of heavy chains paired with λ light chains, while four involved heavy chains paired with κ light chains. For the heavy chains, four distinct IGHV gene segments (IGHV1-4, IGHV1S2, IGHVS5, and IGHV1-15), two IGHD segments (IGHD1 and IGHD2), and a single IGHJ segment (IGHJ5) were identified, resulting in seven unique VDJ rearrangement combinations. The λ light chains utilized four IGLV gene segments (IGLV3-3, IGLV8-13, IGLV8-19, and IGLV8-10) with a solitary IGLJ2 segment, generating four distinct VJ rearrangements, whereas the kappa light chains exhibited only one VJ combination, involving IGKV1-11 and IGKJ2. Furthermore, we classified the 11 anti-E^rns^ mAbs into subtypes based on the heavy chain constant region. The results showed that, except for the E0S9, which belongs to the IGHG-2b subtype, the remaining 10 strains all belong to the IGHG-1 subtype. These results demonstrate that our primer set provides comprehensive coverage, enabling the amplification of a broad spectrum of monoclonal antibodies. Furthermore, the V(D)J gene usage identified aligns with established patterns reported for porcine antibodies [21].

### 2.3. Expression and Reactivity of Porcine Monoclonal Antibodies

**Figure 3:**
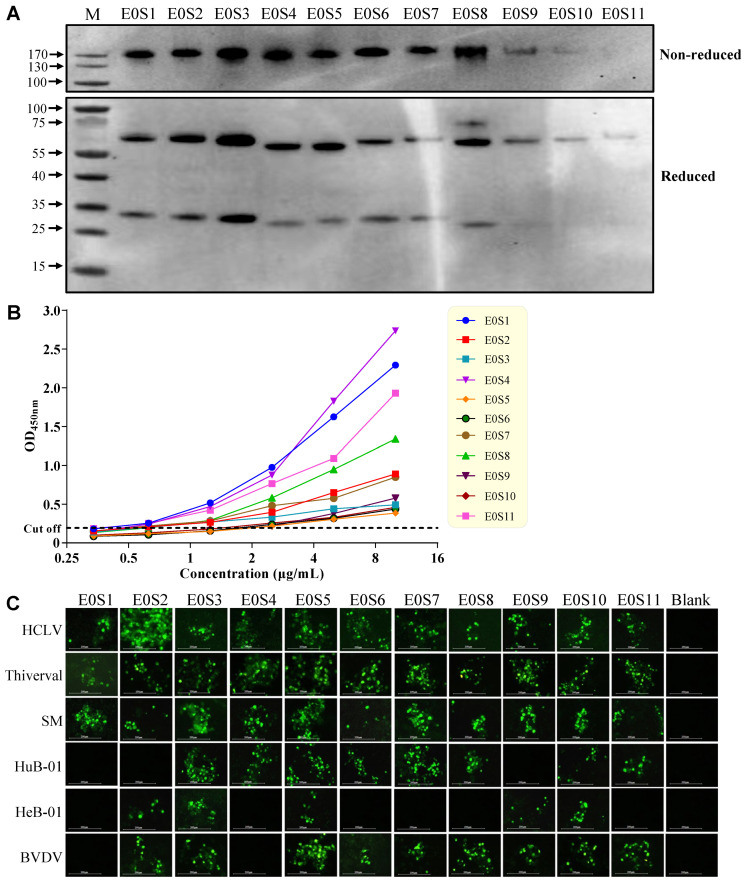
Expression and reactivity of porcine Anti-E^rns^ mAbs. (**A**) Western blot analysis. Purified mAbs were analyzed under non-reducing (upper panel) and reducing (lower panel) conditions. Bands at approximately 160 kDa correspond to the intact antibody, while bands at ~50 kDa and ~25 kDa correspond to the heavy chain (HC) and light chain (LC), respectively. (**B**) Antigen-binding reactivity by indirect ELISA. 96-well plates were coated with purified CSFV E^rns^ protein at 100 ng/well in a carbonate–bicarbonate coating buffer (pH 9.6) at 4 °C overnight. The purified antibody for testing was diluted to 10 μg/mL and serially diluted in a 2-fold ratio. CSFV E^rns^ positive serum and SPF pig serum were used as positive and negative controls, respectively. Rabbit anti-porcine IgG-HRP antibody (1:5000 dilution) was used as a secondary antibody. Absorbance values at the OD_450nm_ wavelength were read using a microplate reader. (**C**) Cross-reactivity profile by immunofluorescence assay (IFA). The reactivity of mAbs was tested against various CSFV strains (subgenotypes 1.1, 2.1 and 2.2) and BVDV. Green fluorescence indicates positive recognition. Scale bar: 100 μm.

All 11 paired mAbs (E0S1–E0S11) were successfully expressed in HEK-293T cells. To confirm the proper assembly and secretion of full-length porcine mAbs, 11 mAbs were purified from the culture supernatant by Ni-column affinity chromatography and detected by Western blot. As expected, under non-reducing conditions, a band of approximately 160 kDa corresponding to the intact antibody was observed. In contrast, under reducing conditions, distinct bands representing the heavy chains (~50 kDa) and light chains (~25 kDa) were observed, confirming the expected disassembly of the antibody structure (Figure 3A). The antigen-binding reactivity of 11 mAbs was evaluated using an indirect ELISA with CSFV E^rns^ protein as the coating antigen. All mAbs exhibited positive reactivity, with E0S1, E0S4, and E0S11 showing the strongest signals, suggesting high affinity (Figure 3B).

Furthermore, the virus reactivity spectrum of the mAbs was assessed by immunofluorescence assay (IFA) against a panel of viruses, which included CSFV subgenotypes 1.1 (HCLV, Thiverval, SM), 2.1 (HuB-01), 2.2 (HeB-01), and bovine viral diarrhea virus (BVDV) (Figure 3C; Table 3). All mAbs recognized subgenotype 1.1 strains (HCLV, Thiverval, and SM). Among them, E0S3, E0S5, and E0S10 demonstrated broad cross-reactivity with all tested CSFV isolates and BVDV, while E0S1 and E0S4 were specific to CSFV without BVDV recognition. Notably, E0S1 was restricted to subgenotype 1.1; E0S2 and E0S9 did not react with the subgenotype 2.1 strain (HuB-01); and E0S4, E0S6, E0S7, E0S8, and E0S11 failed to react with the subgenotype 2.2 strain (HeB-01), highlighting variations in epitope recognition.

### 2.4. Development and Validation of a Blocking ELISA for CSFV E^rns^ Antibodies

**Figure 4:**
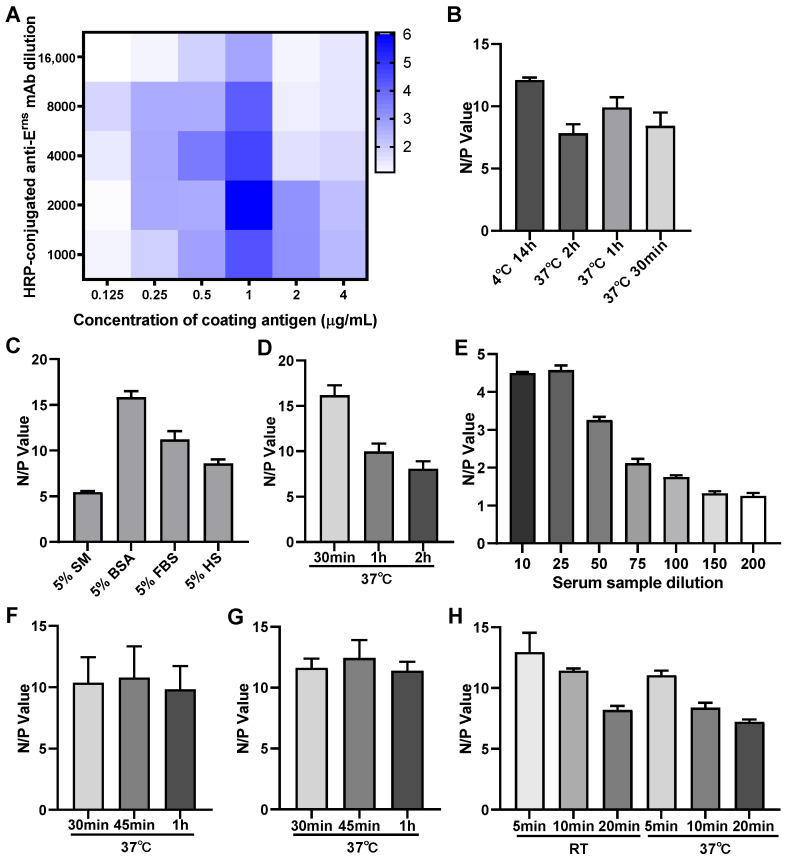
Optimization of the blocking ELISA. (**A**) Determination of the optimal working concentration of coating antigen and mAb-E0S4 by checkerboard titrations. Ratios of negative to positive reference serum (N/P) are presented in a heatmap drawn by the GraphPad Prism software (version 8). The darker the color, the greater the N/P ratio. (**B**) Determination of the optimal antigen incubation conditions. (**C**) Comparison of the blocking effect of four blocking buffers (5% skimmed milk (SM), 5% BSA, 5%FBS, and 5% horse serum (HS)). (**D**) Determination of the optimal incubation time for blocking buffers. (**E**) Determination of the optimal dilution of serum samples. (**F**) Determination of the optimal incubation time for serum samples. (**G**) Determination of the optimal incubation time for HRP-**E^rns^**-mAb E0S4. (**H**) Determination of the optimal chromogenic reaction conditions. All results were presented as the mean ± SD of triplicate experiments.

Based on its strong reactivity and lack of cross-reactivity with BVDV, the porcine mAb E0S4 was selected for HRP conjugation and development of a blocking ELISA. Its blocking efficiency was compared with a murine-derived anti-E^rns^ mAb, E0M11, which we previously prepared [23]. E0S4 demonstrated a significantly higher average N/P (negative-to-positive-serum OD_450nm_ ratio) value of 4.405 compared to 1.985 for E0M11 (Table 4), and was therefore chosen as the detection antibody. Based on the checkerboard titration results, the maximum N/P value was obtained when the E^rns^ coating antigen concentration was 1.0 μg/mL, and the dilution ratio of HRP-conjugated E0S4 mAb was 1:2000 (Figure 4A). It is noteworthy that both excessively high and low concentrations of either the coating antigen or the detection antibody can significantly reduce the N/P value, as they respectively lead to signal saturation or insufficient competition. Thus, the optimal antigen coating concentration and detection antibody dilution of the developed blocking ELISA were determined. Additionally, other important conditions of the blocking ELISA, including antigen coating conditions, blocking buffers and blocking conditions, serum sample dilution and incubation time, HRP-mAb incubation time, and chromogenic reaction conditions, were also optimized. As illustrated in Figure 4, The optimal incubations conditions were: antigen coating at 4 °C for 14 h (Figure 4B), blocking at 37 °C for 2 h (Figure 4C,D), serum sample incubation at 37 °C for 1 h (Figure 4E,F), HRP-mAb incubation at 37 °C for 45 min (Figure 4G), and TMB substrate reaction at room temperature for 5 min (Figure 4H).

### 2.5. Standardization and Determining the Cut-Off Value for Blocking ELISA

**Figure 5:**
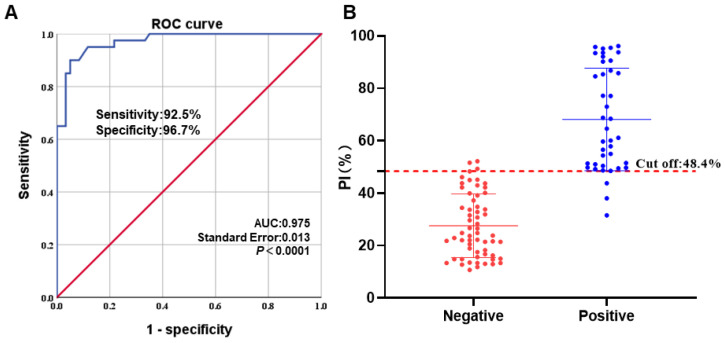
Cut-off value determination, diagnostic sensitivity and specificity analysis of the blocking ELISA. Sixty CSFV-negative serum samples and forty CSFV-positive serum samples were detected by the developed blocking ELISA. (**A**) ROC analysis of blocking ELISA results, while the area under the curve (AUC) of the test was 0.975, and the standard error value is 0.013. (**B**) An interactive dot plot diagram showing the blocking value of serum samples, while the cut-off value was set to 48.4%.

The cut-off value of the developed blocking ELISA was established by testing 40 CSFV-positive sera and 60 CSFV-negative sera samples. A ROC curve statistical analysis was performed to determine the PI cut-off value and to evaluate the diagnostic sensitivity and specificity of the assay (Figure 5A). An interactive dot plot diagram with the PI value of these samples was shown in Figure 5B. According to the ROC analysis, the area under the curve (AUC) was 0.975 (95% confidence interval: 0.950 to 1.000). Furthermore, when the PI cut-off value was set to 48.4% for the developed blocking ELISA, the diagnostic sensitivity and specificity were 92.5% and 96.7%, and the corresponding Youden index (0.925 + 0.967 − 1 = 0.892) was achieved to the maximum (Figure 5A).

### 2.6. Analytic Specificity and Sensitivity of the Blocking ELISA

**Figure 6:**
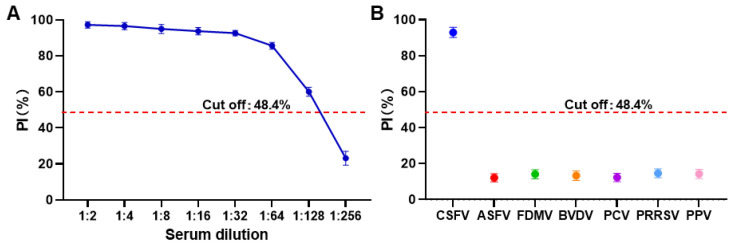
Sensitivity and specificity assay. (**A**) Two–fold serially diluted CSFV-positive reference sera ranging from 1:2 to 1:256 were detected by the blocking ELISA. The cut-off value was marked with a red dashed line. (**B**) Percent of inhibition of the polyclonal anti-sera against various porcine viruses analyzed by the blocking ELISA. Only the CSFV-positive serum showed a higher PI value than the cut-off value. All results were presented as the mean ± SD of triplicate experiments.

The analytical sensitivity of the developed blocking ELISA was determined by testing two-fold serial dilutions (from 1:2 to 1:256) of a CSFV-positive reference serum. The assay could reliably detect antibodies at a maximum serum dilution of 1:128, indicating a satisfactory level of detection sensitivity (Figure 6A). To evaluate analytical specificity, the assay was used to detect several positive reference sera against other viruses, including African swine fever virus (ASFV), foot-and-mouth disease virus (FMDV), BVDV, porcine circovirus (PCV), porcine reproductive and respiratory syndrome virus (PRRSV), and porcine parvovirus (PPV). As shown in Figure 6B, the percent inhibition (PI) values obtained for all these sera were well below the established cut-off value. This result confirms the high specificity of the assay for detecting CSFV antibodies, with no observed cross-reactivity.

### 2.7. Repeatability and Reproducibility of the Blocking ELISA

In the repeatability analysis, three CSFV-positive sera and three CSFV-negative sera were tested with this method on one plate in one run with triplicate. In the reproducibility analysis, these sera were tested in three different plates and three independent runs. As shown in Table 5, the repeatability CV ranged from 1.09% to 4.07%, while the reproducibility CV ranged from 1.64 to 7.84%, indicating good repeatability and reproducibility of the blocking ELISA.

### 2.8. Agreements of the Blocking ELISA with Commercial ELISA Kit

To compare the agreement between the two methods, a total of 56 field sera—including 32 from E2 subunit-vaccinated pigs and 24 from C-strain-vaccinated pigs—were tested in parallel by the developed blocking ELISA and the commercial CSFV E^rns^ indirect ELISA antibody detection kit. As shown in Table 6, the concordance rates of blocking ELISA versus the commercial ELISA kit were 94.6 (53/56). Statistical analysis showed that the Kappa value between the blocking ELISA and the commercial ELISA kit was 0.854, indicating that the established blocking ELISA had a high level of consistency with the commercial kits. Notably, all 32 sera from E2-vaccinated pigs were negative in both assays, underscoring the DIVA compatibility of our test.

## 3. Discussion

Single B cell technology has emerged as a robust platform for generating species-specific monoclonal antibodies (mAbs), overcoming limitations of traditional methods (e.g., hybridoma technology) for generating porcine mAbs [24]. This approach enables the direct amplification of naturally paired antibody genes from the host’s antigen-specific B cells, possessing native conformation and high affinity. Dong et al. [25] successfully obtained two potent neutralizing porcine mAbs against a conserved linear epitope of the CSFV E2 protein by using FACS to isolate antigen-specific single B cells. This work demonstrated the feasibility of this pathway in pigs. Subsequently, Li et al. [21] established a more systematic platform. They sorted single CD45R^+^IgG^+^Ag^+^ B cells from donor pigs with high neutralizing titers against PRRSV and designed primer sets covering all functional V gene segments. Using a two-step PCR, they significantly improved the cloning success rate of antibody genes. More recently, Wang et al. [20] immunized pigs with the CSFV C-strain E2 protein and sorted CE2^+^IgG^+^CD3ε^+^CD8a^−^ single B cells, preparing three porcine mAbs that exhibited neutralizing activity against both vaccine and virulent strains. In this study, we successfully applied single B-cell technology to efficiently generate whole-porcine mAbs against CSFV E^rns^. Using FACS baited with CD21^+^IgM^−^E^rns^-His tag^+^ to obtain CSFV E^rns^-specific B cells while excluding those expressing surface IgM, we isolated 48 antigen-specific single B cells and obtained 11 naturally paired porcine mAbs, underscoring the feasibility of this platform in swine. Importantly, the amplification preserved the native paired structure of the variable regions, which is crucial for maintaining the integrity of the antigen-binding site. Furthermore, our primer design represents a novel feature of this work by allowing for one-step amplification of full-length porcine antibody IgG genes, significantly streamlining the process of antibody cloning and characterization compared to conventional multi-step protocols.

The genetic analysis of 11 anti-E^rns^ mAbs revealed a diverse repertoire, with heavy chains utilizing four IGHV gene segments (IGHV1-4, IGHV1S2, IGHVS5, and IGHV1-15), consistent with findings by Bram et al. [26] that these segments are the most frequently used in pigs. These segments, combined with functional IGHD and IGHJ segments, form seven distinct VDJ rearrangements. Light chains also exhibited diversity, particularly among λ chains, confirming the capability of our method to capture a broad spectrum of the porcine B cell repertoire, which is crucial for identifying mAbs with varied epitope specificities. In addition, the differential usage of light chain types (κ and λ) across species is well-documented. Pigs exhibit a balanced κ:λ ratio of approximately 1:1 [27], whereas cattle show a strong bias toward λ chains (1:19) [28], and the mice and rabbits predominantly use κ chains (19:1) [29]. Accordingly, we designed primers encompassing both κ and λ light chains. The successful amplification of 12 λ and 5 κ chains (κ:λ ≈ 1:2.4) from the sampled B cells validates our primer design. Although this ratio deviates somewhat from the theoretical ~1:1 distribution in swine—a variance likely due to the limited B cell sample size—it demonstrates that our approach reliably captures the porcine antibody repertoire.

A critical finding was the identification of mAbs with distinct and diagnostically valuable reactivity profiles. Three mAbs (E0S3, E0S5, and E0S10) exhibited broad-spectrum reactivity against all tested CSFV genotypes and BVDV, indicating their recognition of conserved epitopes on the E^rns^ protein. More importantly, two porcine mAbs (E0S1, E0S4) demonstrated high specificity for CSFV without cross-reacting with BVDV. This specificity is paramount for developing a CSFV-specific diagnostic assay, effectively addressing the longstanding issue of serological cross-reactivity among *pestiviruses*. Of particular note, mAb E0S1, which is restricted to subgenotype 1.1 strains (HCLV, Thiverval, and SM), can be applied to differentiate between C-strain vaccine immunization and epidemic strain infection. It is noteworthy that the overall reactivity of the porcine mAbs against CSFV E^rns^ protein in IFA appeared superior to that of murine mAbs, which we prepared in parallel [23]. The blocking efficiency of the porcine-derived mAb E0S4 is much higher than that of the mouse-derived mAb E0M11 (Table 4). While these observations are based on a limited comparison, the superior performance of the porcine antibodies is consistent with the hypothesis that host-derived antibodies may possess higher functional affinity. This notion, though requiring validation with a larger panel of antibodies, is an important consideration in infectious disease immunology and diagnostic reagent development.

The selection of mAb E0S4 as the blocking antibody was based on the current CSFV epidemiological landscape in China, where studies demonstrated that genotype 2.1 is the predominant circulating strain, while genotype 1.1 exhibits limited prevalence. In contrast, genotypes 2.2 and 2.3 have essentially disappeared from the circulation [30,31]. Therefore, E0S4’s ability to recognize both the predominant 2.1 and the 1.1 subgenotypes makes it an ideal candidate for a broadly reactive diagnostic assay. The results demonstrated that E0S4 exhibited a significantly higher blocking efficiency, further supporting the observation that host-derived antibodies possess superior binding affinity. Furthermore, the E0S4-based blocking ELISA we established exhibited excellent diagnostic performance, with high sensitivity (92.5%), specificity (96.7%), and no cross-reactivity with other common swine pathogens. The low intra- and inter-assay coefficients of variation (CV < 10%) confirm its robustness and reproducibility. The high concordance (94.6%) with a reference indirect ELISA further validates its reliability for clinical application.

In conclusion, this report is the first to describe the isolation of whole-porcine mAbs against the E^rns^ protein of CSFV from single B cells of a vaccinated pig. We have established a robust platform for generating porcine mAbs by one-step PCR and developed a highly specific and sensitive blocking ELISA for detecting CSFV E^rns^ antibodies. This work provides the swine industry with a reliable tool for monitoring CSFV infection in pigs vaccinated with the E2 subunit vaccine, supporting effective DIVA strategies.

## 4. Materials and Methods

**Animals:** Two healthy Landrace pigs (3 weeks of age) were provided by Beijing Dabeinong Technology Group Co., Ltd. (Beijing, China). The pigs were fed with a standard commercial diet and housed in the Experimental Animal House at China Institute of Veterinary Drug Control (CIVDC). All animal experiments were approved and supervised by the Ethics Committee for Experimental Animal Welfare of the CIVDC.

**Virus Strains, Sera and Cell Lines:** Classical swine fever virus (CSFV) vaccine strains HCLV and Thiverval; CSFV virulent strain Shimen (SM); CSFV epidemic strains HuB-06 (subgenotype 2.1) and HeB-01 (subgenotype 2.2); Bovine viral diarrhea virus (BVDV); HEK-293T, PK-15, and MDBK cell lines; standard positive sera against CSFV, African swine fever virus (ASFV), foot-and-mouth disease virus (FMDV), BVDV, porcine circovirus (PCV), porcine reproductive and respiratory syndrome virus (PRRSV), and porcine parvovirus (PPV), SPF pig serum, CSFV E^rns^ positive serum and clinically immune sera from pigs vaccinated with either the CSFV E2 subunit vaccine or the C-strain vaccine were all preserved by the National/WOAH CSF Reference Laboratory at CIVDC. HEK-293T and MDBK cells were cultured in Dulbecco modified Eagle medium (DMEM; Invitrogen, Carlsbad, CA, USA) supplemented with 10% fetal bovine serum (FBS; PAN-Biotech, Aidenbach, Germany) in a 5% CO_2_ incubator at 37 °C, and PK-15 cells were maintained in modified Eagle medium (MEM; Invitrogen) supplemented with 5% FBS (PAN).

**Animal Immunization:** Two 3-week-old healthy Landrace pigs, negative for both CSFV antigen and antibody, underwent three immunizations on days 0, 21, and 42. For the first and second immunizations, each pig received 10 doses of classical swine fever C-strain vaccine via cervical muscle injection. Forty-two days post vaccination (dpv), a booster immunization with purified CSFV E^rns^ protein was administered at a dose of 100 μg per pig through the same route.

**Isolation of Porcine Peripheral Blood Mononuclear Cells (PBMCs):** Blood samples were collected from the anterior vena of the pig with a higher level of anti-E^rns^ specific antibody response at 52 dpv, then filtered through a 70 μm strainer and diluted 1:1 with PBS. PBMCs were isolated using lymphocyte separation medium according to the manufacturer’s instructions (Solarbio, Beijing, China). After centrifugation, the PBMC layer was resuspended in FACS buffer and diluted to 2 × 10^7^ cells/mL.

**Cell Staining and Sorting of Anti-E^rns^ Specific Single B Cells:** PBMCs were stained by adding a staining cocktail in FACS buffer containing 3 μg His-tagged CSFV E^rns^ protein, 8 μL Alexa Fluor 647-conjugated anti-CD21 antibody, and 2 μL FITC-conjugated anti-pig IgM antibody for 30 min at 4 °C in the dark. Cells were washed and then incubated with 1 μL PE-conjugated anti-His tag secondary antibody for 20 min at 4 °C. After two washes, the cells were resuspended in 500 μL of pre-chilled FACS buffer and sorted for CD21^+^/IgM^−^/E^rns^-His tag^+^ porcine single B cells on a BD FACS Aria II flow cytometer. The target cells were sorted into 96-well PCR plates containing 5 μL of nuclease-free PBS and stored at −80 °C.

**Amplification of Porcine IgG Genes from Single B Cells:** To obtain the full-length immunoglobulin heavy- and light-chain genes (IgH and IgL), the mRNA of sorted porcine single B cells was reverse transcribed into cDNA according to the instructions of the SuperScript™ IV Single Cell/Low Input cDNA PreAmp Kit (Thermo Fisher, Waltham, MA, USA). Based on the available full-length porcine IgG genes in GenBank, we designed degenerate primers. The full-length genes of porcine IgG antibody heavy chain, light chain kappa chain and lambda chain were amplified with these primers according to the instructions of Phusion™ Plus Green PCR Master Mix (Thermo Fisher, USA). All oligonucleotide primers are listed in Table 1. PCR cycling was conducted at 98 °C for 30 s, 35 cycles of 98 °C for 10 s, 60 °C for 10 s, and 72 °C for 50 s (H chain) or 30 s (L chains), and the final extension was at 72 °C for 10 min. The PCR products were checked by 1.5% agarose gel electrophoresis.

**Cloning and Expression of Porcine Monoclonal Antibodies in Mammalian Expression System:** Paired amplicons (H and L chain PCR products) from the same well containing single B cells were purified with MicroElute^®^ Gel Extraction Kit (Omega Bio-Tek, Norcross, GA, USA), digested by restriction enzymes BamHI/AfIII and HindIII, and cloned into the pCDNA3.1-Leader vector. The nucleotide sequences of the heavy (H) and light (L) chain fragments cloned into the vector were verified by DNA sequencing (BGI Genomics, Shenzhen, China). Variable regions were analyzed using the IMGT^®^ database, and constant regions were classified according to the reference [32]. To express monoclonal antibodies (mAbs), each paired H and L chain construct was co-transfected into HEK-293T cells at a mass ratio of 1:2 using PEI MAX (Polysciences, Warrington, PA, USA). Supernatants were collected after 48–72 h. The mAbs were purified by Ni-column affinity chromatography, concentrated with 10 kDa centrifugal filters, quantified by UV absorbance, and stored at −80 °C.

**Identification of Porcine Monoclonal Antibodies:** For Western blot analysis, mAbs were analyzed under reducing and non-reducing conditions by Western blot using anti-His tag antibody. For the indirect ELISA assay, 96-well plates were coated with purified CSFV E^rns^ protein at 100 ng/well in a carbonate–bicarbonate coating buffer (pH 9.6) at 4 °C overnight. The purified antibody for testing was diluted to 10 μg/mL and serially diluted in a 2-fold ratio. SPF pig serum was used as a negative control. Rabbit anti-porcine IgG-HRP antibody (1:5000 dilution) was used as a secondary antibody. Absorbance values at the OD_450nm_ wavelength were read using a microplate reader(Bio-Rad, Hercules, CA, USA). The cut-off value was set at 2.1 times the mean OD_450nm_ of the negative controls. Values above this cut-off were considered positive, and those below were negative.

**Indirect Immunofluorescence Assay (IFA):** To verify the response spectrum of the expressed anti-E^rns^ mAbs to different subtype strains of CSFV (HCLV, SM, HeB-01 and HuB-06) and BVDV, PK-15 and MDBK cells were seeded on 12-well plates and cultured overnight, followed by infection with the above CSFV or BVDV strains at a dose of 200TCID_50_. At 72 h after infection, the cells were fixed with 4% paraformaldehyde, permeabilized with 0.2%Triton X-100, blocked with 1% bovine serum albumin (BSA), and incubated with anti-E^rns^ mAbs, followed by incubation with FITC-conjugated goat anti-pig IgG antibodies (BioLegend, San Diego, CA, USA), and the cells were examined with a fluorescence microscope (Nikon, Tokyo, Japan).

**Development of a Blocking ELISA for CSFV E^rns^ Antibodies:** The purified anti-E^rns^ mAbs were labeled with HRP (Tsingke, Beijing, China) to establish a blocking ELISA for the detection of CSFV antibodies. A checkerboard titration was used to determine the optimal antigen coating concentration of E^rns^ protein and the working concentration of the mAb E0S4. The purified E^rns^ protein was diluted to 0.125, 0.25, 0.5, 1, 2 and 4 μg/mL in carbonated coating buffer (pH = 9.6) and added to wells of a 96-well ELISA plate. The plates were then allowed to coat at 4 °C overnight, washed three times with PBST, and blocked with 5% skim milk in PBS for 2 h at 37 °C. After another three washes with PBST, 100 μL/well of serially diluted CSFV standard positive sera and negative sera (SPF pig serum) were separately added to the well and then incubated for 1 h at 37 °C. After three washes, 100 μL/well of serially diluted HRP-anti-E^rns^ mAb E0S4 in the range of 1:1000–1:16,000 was added to the plate and incubated for 1 h at 37 °C. After washing three times, 100 μL/well of TMB substrate was added to all wells of the plate and incubated for 10 min at room temperature. By adding a stop solution for TMB substrate (Beyotime Biotechnology, Shanghai, China), the chromogenic reaction was stopped. Subsequently, the optical density of each well at 450 nm was measured using a spectrophotometer (Molecular Devices, San Jose, CA, USA). The optimal E^rns^ protein-coating concentration and HRP-anti-E^rns^ mAb working concentration were determined according to the highest OD_450nm_ ratio of negative to positive serum (N/P). Following the determination of the optimal concentrations for the coating antigen and the mAb, we further optimized other critical parameters of the blocking ELISA. These included the antigen coating conditions, suitable blocking buffers and blocking conditions, serum sample dilution and incubation time, HRP-anti-E^rns^ mAb incubation time, and chromogenic reaction conditions. More details about the blocking ELISA are summarized in Table 7.

**Determination of cut-off value:** A total of 100 serum samples (40 CSFV-positive sera and 60 CSFV-negative sera samples) were tested by the optimized blocking ELISA to determine the cut-off value, diagnostic sensitivity, and diagnostic specificity of this method. Raw data were collected and the percent inhibition (PI) values of the test samples were calculated via the following formula: PI = (1 − test sample OD_450_/negative control OD_450_) × 100% [33]. The PI value of each serum was analyzed by the ROC curve using SPSS software (version 26.0) to present the area under the curve (AUC). The cut-off value of each serum corresponding to sensitivity and specificity values was used to calculate the Youden index according to the formula: Youden index = sensitivity + specificity − 1. The cut-off value corresponding to the maximum Youden index is the optimal cut-off point.

**Estimation of the analytic sensitivity and specificity:** CSFV standard positive serum of two-fold serial dilutions between 1:2 and 1:256 was used to evaluate the analytical sensitivity of the method. The highest dilution of the standard positive serum producing a PI value greater than the cut-off point was defined as the analytic sensitivity of the blocking ELISA. Meanwhile, six other swine viruses (CSFV, ASFV, PRRSV, FMDV, PCV, BVDV, and PPV) with standard positive serum were used to estimate the analytical specificity of the blocking ELISA.

**Estimation of the repeatability and reproducibility:** The repeatability of the blocking ELISA was analyzed by using three positive serum samples with different antibody levels and three negative serum samples in triplicate wells in one run. Meanwhile, the reproducibility of the method was estimated by three runs at different times. The coefficient of variation (CV) was used to quantify the degree of variation in the blocking ELISA using the following formula: CV = standard deviation (SD)/mean PI value of each serum sample.

**Clinical Sample Testing:** Fifty-six clinical serum samples were tested using the developed blocking ELISA and a commercial CSFV E^rns^ antibody indirect ELISA kit (Luoyang Putai, Luoyang, China). The degree of agreement (Kappa value) of the blocking ELISA with the commercial ELISA kit was calculated using SPSS software (version 26.0). The higher the kappa value, the better the consistency between the two methods; if the kappa value is between 0 and 0.40, the consistency between the two methods is poor; if the kappa value reaches 0.75 or above, it indicates that the consistency between the two methods is high.

## Figures and Tables

**Table 1:** Primer sets to amplify the whole coding regions of porcine IgG chains.

Primer Name	Sequence (5′-3′)	PCR Product
Pig-His-HF	AAGCTTGCCGCCACCATGGAGTTTCGGCTGAACTGG	whole H chain
Pig-His-HR	GGATCCCTA**GTGGTGATGGTGATGATG**TTTACCCGGAGTCTTGGAGATAGAC	
Pig-His-λF	AAGCTTGCCGCCACCATGGCCTGGACGGTGCT	whole λ chain
Pig-His-λR	GGATCCTCA**GTGGTGATGGTGATGATG**GGCGCACTCGGAGGGC	
Pig-His-κF	CTTAAGGCCGCCACCATGAGGGCCCCCATGCACCTCCTTG	whole κ chain
Pig-His-κR	GGATCCTCA**GTGGTGATGGTGATGATG**AGCCTCACACTCGTTCCTGTTGAAGCT	

Note: The restriction enzyme sites for BamHI, AflII, and HindIII are underlined. The His-tag sequence is shown in bold.

**Table 2:** The gene information of porcine mAbs against the CSFV E^rns^ protein.

mAbs	Heavy Chain			Light Chain		Subtypes
	V Gene	D Gene	J Gene	V Gene	J Gene	
E0S1	V1S2*01	D1*01	J5*01	V3-3*01	J2*01	IGHG-1
E0S2	V1-4*01	D1*01	J5*01	V3-3*01	J2*01	IGHG-1
E0S3	V1S2*01	D2*01	J5*01	V8-13*01	J2*01	IGHG-1
E0S4	V1-4*02	D2*01	J5*01	V8-19*02	J2*01	IGHG-1
E0S5	V1-4*02	D1*01	J5*01	V8-10*01	J2*01	IGHG-1
E0S6	V1-15*01/S2*01	D2*01	J5*01	V1-11*02	J2*01	IGHG-1
E0S7	V1S2*01	D1*01	J5*01	V1-11*02	J2*01	IGHG-1
E0S8	VS5*01	D1*02	J5*01	V3-3*01	J2*01	IGHG-1
E0S9	V1-4*02	D2*01	J5*01	V1-11*02	J2*01	IGHG-2b
E0S10	V1S2*01	D1*01	J5*01	V1-11*02	J2*01	IGHG-1
E0S11	V1-4*01	D1*01	J5*01	V3-3*01	J2*01	IGHG-1

Note: * indicates allelic variants.

**Table 3:** The reactivity spectrum of 11 Anti-E^rns^ mAbs.

mAbs	ELISA	IFA					
		HCLV (1.1)	Thiveral (1.1)	SM (1.1)	HuB-06 (2.1)	HeB-01 (2.2)	BVDV
E0S1	+	+	+	+	-	-	-
E0S2	+	+	+	+	-	+	+
E0S3	+	+	+	+	+	+	+
E0S4	+	+	+	+	+	-	-
E0S5	+	+	+	+	+	+	+
E0S6	+	+	+	+	+	-	+
E0S7	+	+	+	+	+	-	+
E0S8	+	+	+	+	+	-	+
E0S9	+	+	+	+	-	+	+
E0S10	+	+	+	+	+	+	+
E0S11	+	+	+	+	+	-	+

Note: +: reactivity; -: no reactivity.

**Table 4:** Comparative efficacy of blocking antibodies: porcine E0S4 vs. murine E0M11.

mAbs	OD_450nm_		N/P	Average N/P
	Positive Serum Sample	Negative Serum Sample		
E0S4	0.590	2.624	4.447	4.405
	0.576	2.523	4.380	
	0.577	2.531	4.386	
E0M11	1.227	2.433	1.983	1.985
	1.295	2.562	1.978	
	1.267	2.527	1.994	

**Table 5:** Repeatability and reproducibility analyses of the blocking ELISA.

Samples	Repeatability			Reproducibility		
	Mean	SD	CV (%)	Mean	SD	CV (%)
Strong–positive	91.70%	0.010	1.09%	91.50%	0.015	1.64%
Medium–positive	86.50%	0.011	1.27%	84.60%	0.020	2.36%
Weak–positive	71.30%	0.022	3.09%	71.20%	0.038	5.34%
Negative 1	17.20%	0.007	4.07%	15.30%	0.012	7.84%
Negative 2	15.40%	0.005	3.25%	14.50%	0.009	6.21%
Negative 3	20.60%	0.008	3.88%	18.40%	0.014	7.61%

**Table 6:** Comparison results of blocking ELISA and commercial ELISA kits for detecting field samples.

The Blocking ELISA	Commercial ELISA Kit		Total	Agreement (%)	Kappa Value
	+	-			
+	12	1	13	94.6% (53/56)	0.854
-	2	41	43		
Total	14	42	56		

Note: “+” indicates a positive sample; “-” indicates a negative sample.

**Table 7:** The optimization of react condition of CSFV blocking ELISA.

Blocking ELISA Method	Concentration/Dilution	Reaction Conditions
Antigen Coating	0.125, 0.25, 0.5, 1, 2 and 4 μg/mL	37 °C 30 min, 37 °C 1 h, 37 °C 2 h and 4 °C 14 h
Blocking Conditions	5% skim milk, 5%BSA, 5%FBS, and 5% horse serum	37 °C 30 min, 37 °C 1 h and 37 °C 2 h
Serum sample	1:10, 1:25, 1:50, 1:75, 1:100, 1:150, 1:200	37 °C 30 min, 37 °C 45 min and 37 °C 1 h
HRP Labeled Antibody	1:1000, 1:2000, 1:4000, 1:8000, 1:16,000	37 °C 30 min, 37 °C 45 min and 37 °C 1 h
TMB Substrate	/	37 °C and room temperature (25 °C) for 5 min, 10 min and 15 min, respectively

## Data Availability

The datasets for the current study are available from the corresponding author on reasonable request.
